# Geological Survey

**Published:** 1835

**Authors:** 


### XII.—Geological Surveys.

Several of our state legislatures have, within the last few years, made provision for geological surveys of their respective territories; and some of them have been finished, while others are in progress. This is a policy which we hope, ere long, to see adopted by every state in the Union. As citizens and men of science, we feel a deep interest in such surveys; and as physicians we desire to see them executed. It has long been known, that some diseases are peculiar to certain countries, and that others are strikingly modified by soil and water, not less than by climate. A geological survey is the basis of all medical topography; and until such a survey of the different states is made by competent persons, there will be no safe and solid foundation for their medical history. It is by these examinations that we acquire a knowledge of the chemical constitution of the soil, and its substrata, and their influence on the waters of wells, springs, and streams; the extent and composition of alluvial and diluvial deposites; the elevation of the whole country above the level of the sea; its depression below the adjoining mountains, and the height of its hills over the intervening plains; the distance and bearing of the alpine ranges which modify its temperature, and the course of its valleys and gorges, which exert so much influence on the direction and velocity of its winds. With every survey of this kind there should be connected, to render it beneficial in the highest degree to medical science, a flora of its medicinal plants, many of which are limited to particular soils and elevations.

In the west the state of Tennessee, as we announced in a preceding number, has taken the lead in this particular application of science; but we are happy in being able say, that Kentucky, Indiana and Ohio are likely to follow her example; and would exhort our readers to use their influence with those to whom this subject is confided, till it shall be fully accomplished.

